# Asperuloside and Asperulosidic Acid Exert an Anti-Inflammatory Effect via Suppression of the NF-κB and MAPK Signaling Pathways in LPS-Induced RAW 264.7 Macrophages

**DOI:** 10.3390/ijms19072027

**Published:** 2018-07-12

**Authors:** Jingyu He, Xianyuan Lu, Ting Wei, Yaqian Dong, Zheng Cai, Lan Tang, Menghua Liu

**Affiliations:** 1 Bioengineering Research Centre, Guangzhou Institute of Advanced Technology, Chinese Academy of Sciences, Guangzhou 511458, China; jy.he@giat.ac.cn (J.H.); ting.wei@siat.ac.cn (T.W.); 2 Guangdong Provincial Key Laboratory of New Drug Screening, School of Pharmaceutical Sciences, Southern Medical University, Guangzhou 510515, China; luxianyuan_211723@163.com (X.L.); yqdongchn@163.com (Y.D.); caizheng2002@sina.com (Z.C.); tl405@smu.edu.cn (L.T.)

**Keywords:** iridoids, nuclear factor-kappaB, mitogen-activated protein kinase, anti-inflammation

## Abstract

*Hedyotis diffusa* is a folk herb that is used for treating inflammation-related diseases in Asia. Previous studies have found that iridoids in *H. diffusa* play an important role in its anti-inflammatory activity. This study aimed to investigate the anti-inflammatory effect and potential mechanism of five iridoids (asperuloside (ASP), asperulosidic acid (ASPA), desacetyl asperulosidic acid (DAA), scandoside methyl ester (SME), and *E*-6-*O*-*p*-coumaroyl scandoside methyl ester (CSME)) that are presented in *H. diffusa* using lipopolysaccharide (LPS)—induced RAW 264.7 cells. ASP and ASPA significantly decreased the production of nitric oxide (NO), prostaglandin E_2_ (PGE_2_), tumor necrosis factor-α (TNF-α), and interleukin-6 (IL-6) in parallel with the inhibition of inducible nitric oxide synthase (iNOS), cyclooxygenase-2 (COX-2), TNF-α, and IL-6 mRNA expression in LPS-induced RAW 264.7 cells. ASP treatment suppressed the phosphorylation of the inhibitors of nuclear factor-kappaB alpha (IκB-α), p38, extracellular signal-regulated kinase (ERK), and c-Jun N-terminal kinase (JNK). The inhibitory effect of ASPA was similar to that of ASP, except for p38 phosphorylation. In summary, the anti-inflammatory effects of ASP and ASPA are related to the inhibition of inflammatory cytokines and mediators via suppression of the NF-κB and mitogen-activated protein kinase (MAPK) signaling pathways, which provides scientific evidence for the potential application of *H. diffusa*.

## 1. Introduction

Inflammation is a natural defense response against bacteria, viral, and fungal infections [1]. During the development of inflammation, macrophages can be recruited to inflammatory sites and play an essential role by stimulating intracellular cascades of cytokines and chemokines via various signals [2,3]. Lipopolysaccharide (LPS) is one of the most common inflammatory triggers derived from Gram-negative bacteria. It can cause multiple inflammatory reactions by activating macrophages [4] and results in the increase of inflammatory mediators, such as nitric oxide (NO) and prostaglandin E_2_ (PGE_2_), and inflammatory cytokines, such as tumor necrosis factor-α (TNF-α) and interleukin-6 (IL-6) [5,6,7]. There is good evidence that nuclear factor-kappaB (NF-κB) and mitogen-activated protein kinases (MAPKs) are two crucial signaling pathways in the inflammatory process induced by LPS [5,8,9]. Once activated, the gene expression of pro-inflammatory cytokine is induced collaboratively by NF-κB and MAPKs, subsequently releasing cytokines under inflammatory conditions [10]. Therefore, chemicals targeting NF-κB and/or MAPK signaling pathways are supposed to be anti-inflammatory candidates for the treatment of inflammation related disorders.

**Figure 1:**
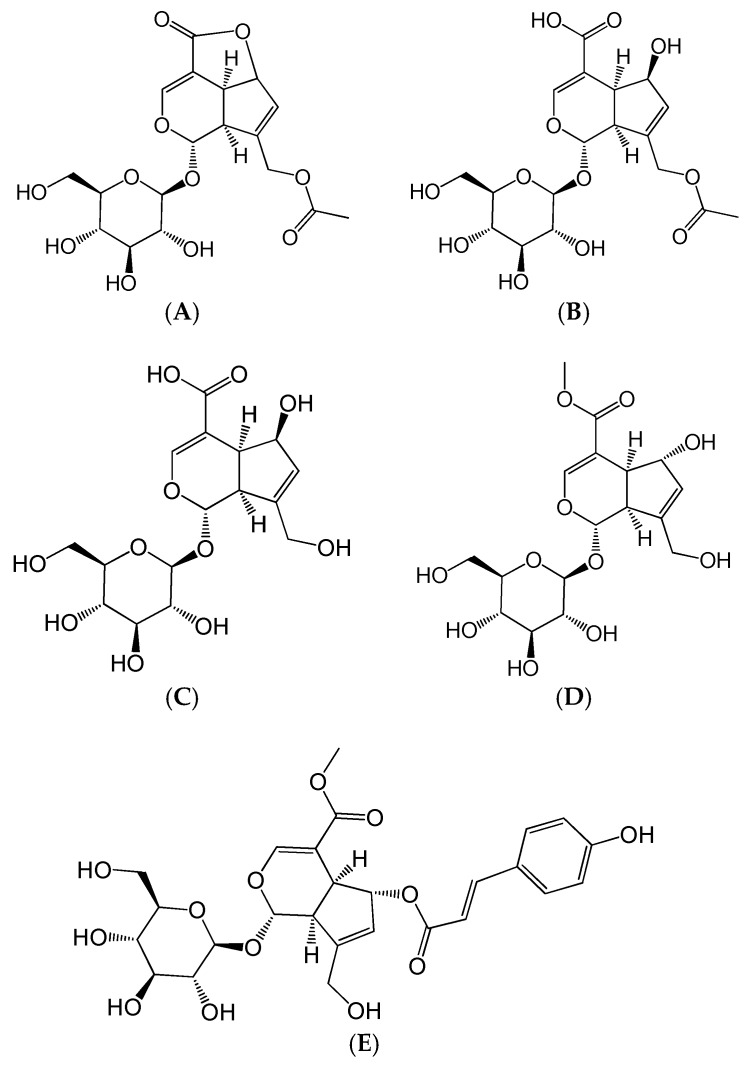
Chemical structures of the five iridoids. (**A**) Asperuloside (ASP); (**B**) Asperulosidic acid (ASPA); (**C**) Desacetyl asperulosidic acid (DAA); (**D**) Scandoside methyl ester (SME); and (**E**) *E*-6-*O*-*p*-Coumaroyl scandoside methyl ester (CSME).

*Hedyotis diffusa* Willd (*Rubiaceae*) is a famous folk herb that is widely distributed in China, Indonesia, Nepal, and other Asian regions [11]. Generally, *H. diffusa* is used as a single herb or in Chinese traditional medicine prescription for the treatment of nephritis, arthritis, bronchitis, and appendicitis [12]. Modern pharmacological studies have confirmed that *H. diffusa* possesses multiple effects, such as anti-inflammatory, anti-cancer, neuroprotective, hepatoprotective, and immunemodulating activities [13]. Iridoids, flavonoids, and anthraquinones are the main constituents responsible for the multiple bioactivities of *H. diffusa* [14,15]. Scandoside, an iridoid isolated from *H. diffusa*, has been proven to be an anti-inflammatory compound [16]. Previously, we confirmed that the aqueous extract of *H. diffusa*, mainly containing asperuloside (ASP), asperulosidic acid (ASPA), desacetyl asperulosidic acid (DAA), scandoside methyl ester (SME), and *E*-6-*O*-*p*-coumaroyl scandoside methyl ester (CSME), showed a protective effect on LPS-induced renal inflammation in mice (Figure 1) [17]. However, the contribution of these five iridoids to the anti-inflammatory effect and their anti-inflammatory mechanisms is still unclear.

In this study, the anti-inflammatory effect of five iridoids found in *H. diffusa* was investigated using LPS-induced RAW 264.7 cells. The underlying mechanisms of the anti-inflammatory compounds were further illustrated.

## 2. Results

### 2.1. Effects of Five Iridoids on RAW 264.7 Cell Viability

**Figure 2:**
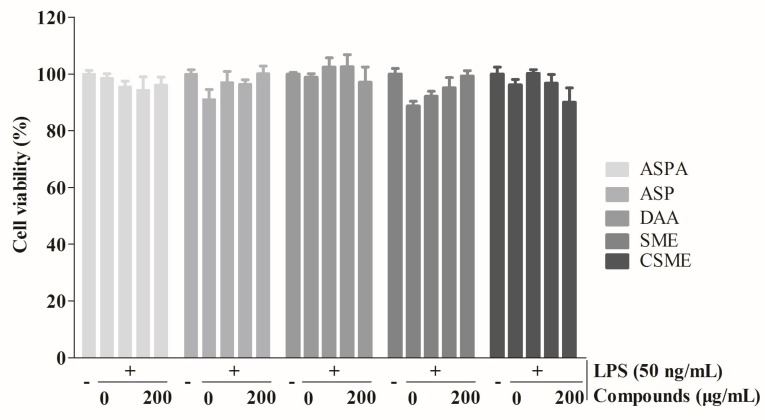
Effects of five iridoids on the viability of RAW 264.7 cells. RAW 264.7 cells were treated with asperuloside (ASP), asperulosidic acid (ASPA), desacetyl asperulosidic acid (DAA), scandoside methyl ester (SME) and *E*-6-*O*-*p*-coumaroyl scandoside methyl ester (CSME) at the concentration of 0, 50, 100, and 200 μg/mL, respectively, for 1 h, and then induced with 50 ng/mL lipopolysaccharide (LPS) for 24 h. Cell viability was measured by the Cell Counting Kit-8 (CCK-8) assay (*n* = 3).

As shown in Figure 2, the percentages of cell viability for the five iridoids were from 94.83 to 105.52%. Cell viability was not significantly affected by the five iridoids at various concentrations (0–200 μg/mL) after 24 h of treatment in the presence of 50 ng/mL LPS. These data indicated that ASP, ASPA, DAA, SME, and CSME had no toxic effect on RAW 264.7 cells at concentrations below 200 μg/mL. Subsequent experiments were performed at the concentrations of 40, 80, and 160 μg/mL.

### 2.2. Effects of Five Iridoids on Inflammatory Mediators and Inflammatory Cytokines in RAW 264.7 Cells

**Figure 3:**
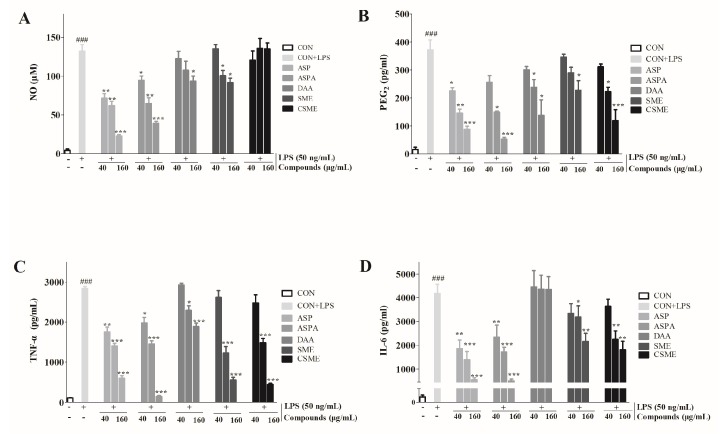
Effects of five iridoids on the productions of nitric oxide (NO), prostaglandin E_2_ (PGE_2_), tumor necrosis factor-α (TNF-α), and interleukin-6 (IL-6). RAW 264.7 cells were treated with ASP, ASPA, DAA, SME, and CSME at the concentration of 40, 80, and 160 μg/mL, respectively, for 1 h, and then induced with 50 ng/mL LPS for 24 h. The levels of NO (**A**), PGE_2_ (**B**), TNF-α (**C**), and IL-6 (**D**) in the cell-free culture were measured by ELISA. * *p* < 0.05, ** *p* < 0.01 and *** *p* < 0.001 versus LPS-only treatment group; ^###^ *p* < 0.001 versus control group (*n* = 3).

As shown in Figure 3, the levels of inflammatory mediators (NO and PGE_2_) and inflammatory cytokines (TNF-α and IL-6) in the LPS-treatment group were significantly increased when compared with the control group. However, the groups treated with the five iridoids showed different behaviors. ASP and ASPA treatment significantly reduced the level of NO (*p* < 0.05), whereas no significant difference was observed in the CSME group at any concentration. The effects on NO in the DAA- and SME-treated groups were only found at higher concentration levels. Furthermore, all iridoids, except SME, inhibited the production of PGE_2_ and TNF-α at 80 and 160 μg/mL (*p* < 0.05). In addition, ASP and ASPA treatment significantly decreased the level of IL-6 in concentration-dependent manners. SME and CSME treatment significantly reduced the production of IL-6 at 80 and 160 μg/mL. Conversely, no inhibitory effect of DAA on the production of IL-6 was observed. Considering the strongly potential bioactivities, ASP and ASPA were selected for further investigation.

### 2.3. Effects of ASP and ASPA on TNF-α and IL-6 mRNA Expression in LPS-Induced RAW 264.7 Cells

**Figure 4:**
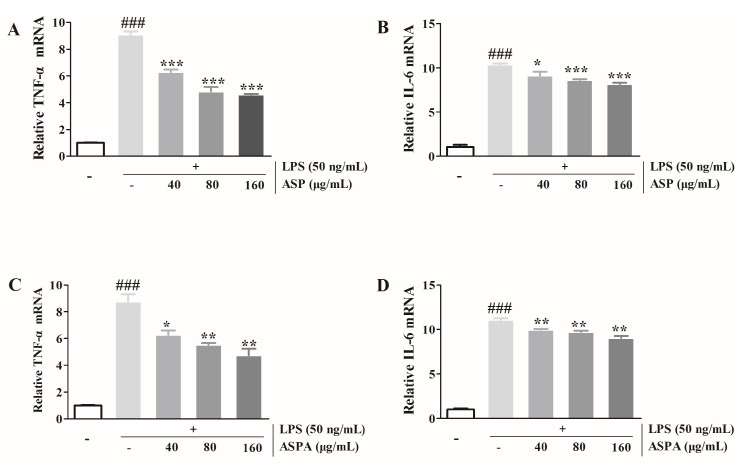
Effects of ASP and ASPA on TNF-α and IL-6. RAW 264.7 cells were treated with ASPA and ASP (40, 80, and 160 μg/mL) for 1 h and then induced with LPS (50 ng/mL) for 24 h. The TNF-α and IL-6 mRNA were analyzed by real-time PCR. (**A**) The TNF-α levels in ASP treatment groups. (**B**) The IL-6 levels in ASP treatment groups. (**C**) The TNF-α mRNA levels in ASPA treatment groups. (**D**) The IL-6 levels in ASPA treatment groups. * *p* < 0.05, ** *p* < 0.01 and *** *p* < 0.001 versus LPS-only treatment group; ^###^ *p* < 0.001 versus control group (*n* = 3).

The mRNA expression of TNF-α and IL-6 was tested to determine whether ASP and ASPA regulated their transcriptional levels. As shown in Figure 4, ASP and ASPA treatment significantly down-regulated the mRNA levels of TNF-α and IL-6 in LPS-induced RAW 264.7 cells compared with the group treated with LPS alone. Similarly, the protein levels of TNF-α and IL-6 were also reduced by treatment with ASP and ASPA.

### 2.4. Effects of ASP and ASPA on iNOS and COX-2 Protein and mRNA Expression in LPS-Induced RAW 264.7 Cells

**Figure 5:**
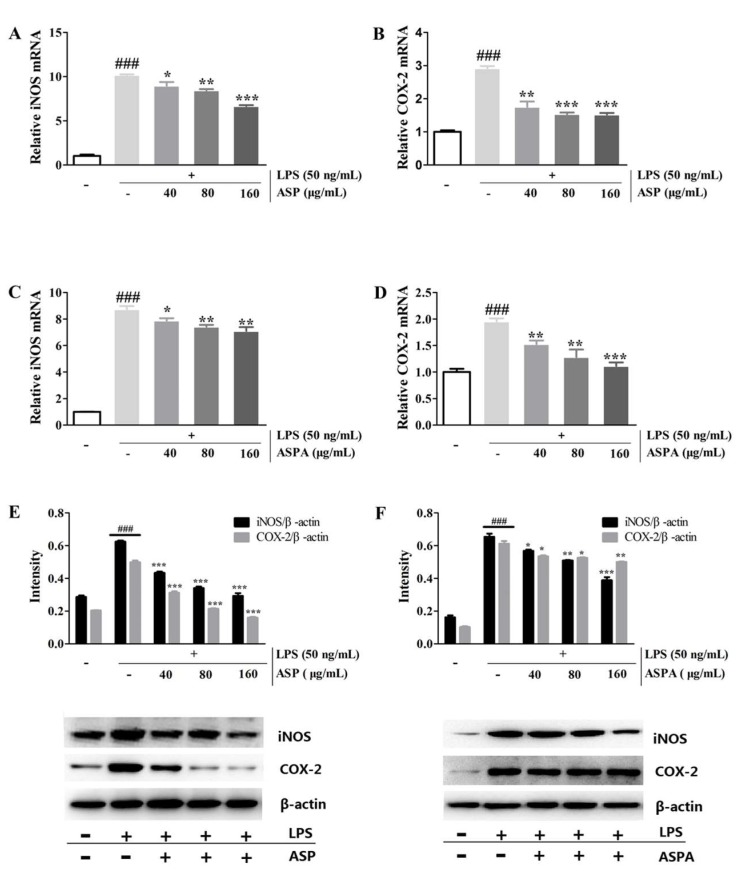
Effects of ASP and ASPA on inducible nitric oxide synthase (iNOS) and cyclooxygenase-2 (COX-2) mRNA and protein expression. RAW 264.7 cells were treated with ASP and ASPA (40, 80, and 160 μg/mL) for 1 h and then induced with LPS (50 ng/mL) for 24 h. mRNA levels were analyzed by real-time PCR. (**A**) The levels of iNOS mRNA in ASP treatment groups. (**B**) The levels of COX-2 mRNA in ASP treatment groups. (**C**) The levels of iNOS mRNA in ASPA treatment groups. (**D**) The levels of COX-2 mRNA in ASPA treatment groups. The proteins were analyzed by Western blot. The quantitative evaluation of protein band by densitometry was shown. (**E**) The expression levels of iNOS and COX-2 in ASP treatment groups. (**F**) The expression levels of iNOS and COX-2 in ASPA treatment groups. * *p* < 0.05, ** *p* < 0.01 and *** *p* < 0.001 versus LPS-only treatment group; ^###^ *p* < 0.001 versus control group (*n* = 3).

To investigate whether ASP and ASPA suppressed NO and PGE_2_ via inhibition of their corresponding synthases, the protein and mRNA expression of inducible nitric oxide synthase (iNOS) and cyclooxygenase-2 (COX-2) was measured. LPS induced a significant up-regulation of the mRNA transcript levels of iNOS and COX-2, while ASP and ASPA treatment significantly down-regulated their mRNA transcript levels, in a concentration-dependent manner (Figure 5A–D). Western blot analysis showed that ASP and ASPA treatment reduced the protein levels of iNOS and COX-2 induced by LPS in a concentration-dependent manner (Figure 5E,F). The reduction of iNOS and COX-2 mRNA and protein levels correlated with the reduced production of NO and PGE_2_, respectively.

### 2.5. Effects of ASP and ASPA on NF-κB and MAPK Pathways in LPS-Induced RAW 264.7 Cells

**Figure 6:**
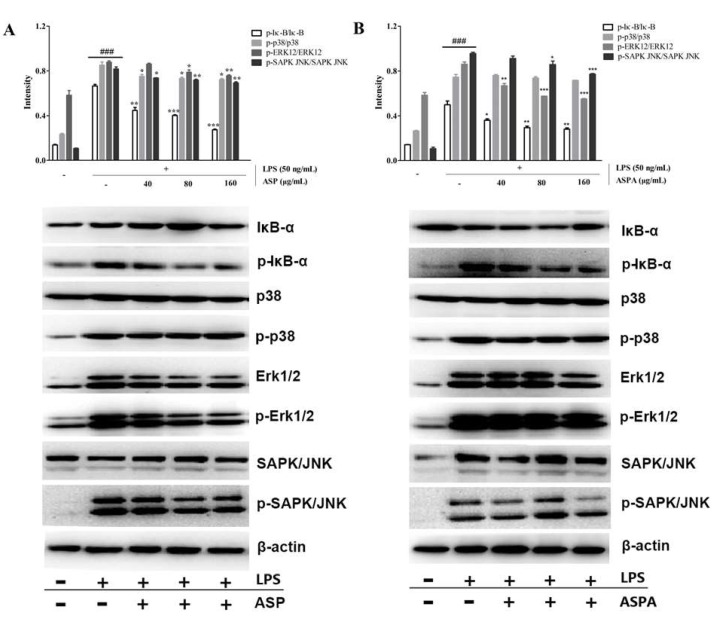
Effects of ASP and ASPA on nuclear factor-kappaB alpaha (IκBα), p38, extracellular signal-regulated protein kinases 1/2 (Erk1/2), and stress-activated protein kinase (SAPK)/c-Jun N-terminal kinase (JNK) phosphorylation. RAW 264.7 cells were treated with ASP and ASPA (40, 80, and 160 μg/mL) for 1 h and then induced with LPS (50 ng/mL) for 24 h. The protein was analyzed by Western blot. The quantitative evaluation of protein bands by densitometry is shown. (**A**) The phosphorylation levels of IκBα, p38, Erk1/2, and SAPK/JNK in ASP treatment groups. (**B**) The phosphorylation levels of IκBα, p38, Erk1/2, and SAPK/JNK in ASPA treatment groups. * *p* < 0.05, ** *p* < 0.01 and *** *p* < 0.001 versus LPS-only treatment group; ^###^ *p* < 0.001 versus control group (*n* = 3).

To study the potential anti-inflammatory mechanism, we examined the effects of ASP and ASPA on nuclear factor-kappaB alpaha (IκB-α) phosphorylation and degradation in LPS-induced RAW 264.7 cells. As shown in Figure 6, LPS-induced IκB-α phosphorylation was significantly decreased after pretreatment with ASP and ASPA in a concentration-dependent manner. The potential involvement of MAPKs was also investigated by testing the modulatory effects of ASP and ASPA on MAPK signaling pathways. In LPS-induced RAW 264.7 cells, p38, extracellular signal-regulated protein kinases 1/2 (Erk1/2), and c-Jun N-terminal kinase (JNK) were triggered high phosphorylation, whereas the phosphorylation of p38, Erk1/2, and JNK was inhibited by ASP in a concentration-dependent manner. For the effect of ASPA on MAPKs, ASPA treatment decreased Erk1/2 phosphorylation at all concentration levels, but there was no effect on p-p38.

## 3. Discussion

To date, numerous natural products have been identified as potential anti-inflammatory agents that can scavenge inflammatory mediators [18,19,20,21,22]. Among them, iridoids play an important role in inflammatory treatment and are considered as the major bioactive constituents of *H. diffusa* [16,17]. In a phytochemical analysis, ASP, ASPA, DAA, SME, and CSME were found by ultra performance liquid chromatography-electrospray ionization-quadrupole-time of flight-mass spectrometry (UPLC-ESI-Q-TOF-MS) methods and identified by comparison with their reference substances [17]. Moreover, these five iridoids were also found in various medicinal plants [23,24,25]. However, there is still a lack of further pharmacological studies on these iridoids, especially their anti-inflammatory mechanism. In this study, the results indicated that the five iridoids could inhibit inflammation related factors at particular concentrations. Importantly, ASP and ASPA were the target compounds that had the greatest anti-inflammatory effects among the five compounds.

NO is one of the most important inflammatory mediators. When activated by inflammatory inducers, the immune cells will produce NO, which is catalyzed by iNOS [26,27]. A high concentration of NO can cause oxidative damage or even inflammatory and autoimmune diseases [28,29]. COX enzymes produce prostaglandins involved in the inflammatory process. Actually, two distinct cyclooxygenase isoforms, namely COX-1 and COX-2, are commonly used as molecular targets for non-steroidal anti-inflammatory drugs (NSAIDs) [30]. COX-1 is expressed ubiquitously and constitutively, which plays a housekeeping role in processes, such as gastrointestinal mucosa protection, while COX-2 is not constitutively expressed in the cell, but can be activated by inducers, such as endotoxins, cytokines, and growth factors [31]. In LPS-induced macrophages, COX-2 can convert arachidonic acid to PGG_2_ and, finally, to PGE_2_ [32,33,34]. Selective inhibition of COX-2 expression can block PGE_2_ production induced by inflammation and alleviate inflammation. Thus, COX-2 is regarded as a target for anti-inflammatory treatment. The results demonstrated that ASP and ASPA significantly decreased the levels of NO and PGE_2_ by suppressing iNOS and COX-2, respectively. Moreover, pro-inflammatory cytokines, such as TNF-α and IL-6, play crucial roles in the development of inflammatory diseases and are involved in the innate immunity and autoimmune diseases [35,36]. The levels of inflammatory-related factors are considered to be indicators of the degree of inflammation. Thus, the expression of TNF-α and IL-6 mRNA in LPS-induced RAW 264.7 cells was measured. ASPA and ASP significantly suppressed TNF-α and IL-6 mRNA expression. These results suggested that ASPA and ASP exerted an anti-inflammatory effect through the inhibition of iNOS, COX-2, TNF-α, and IL-6 mRNA expression.

NF-κB is a key signaling pathway that stimulates the expression of inflammatory mediators and cytokines, including iNOS, COX-2, TNF-α, and IL-6 [5,9]. The IκBα proteins are phosphorylated and degraded, leading to activation of NF-κB, which triggers the transcription of inflammatory-related factors [37]. When activated, p65 translocates into the nucleus and then promotes the transcription of corresponding pro-inflammatory genes [38,39]. ASP and ASPA were found to significantly inhibit the NF-κB activation via decreasing the phosphorylation of IκB. MAPKs, including ERK, JNK, and p38, also take part in the regulation of the expression of inflammation-related genes, leading to the overproduction of pro-inflammatory cytokines [40,41]. Western blot analysis showed that ASP inhibited LPS-induced phosphorylation of p38, ERK, and JNK in RAW 264.7 cells, thus, displaying an anti-inflammatory effect consistent with a previous report [42]. ASPA also exerted an anti-inflammatory effect via suppression of the MAPK signaling pathway, though it did not inhibit the phosphorylation of p38. This data demonstrated that the anti-inflammatory mechanisms of ASP and ASPA were likely via the inhibition of NF-κB and MAPK signaling pathways. The results also indicated that ASPA and ASP were the main compounds responsible for the anti-inflammatory effect of *H. diffusa*. Given the relationship between inflammation and tumors [43], the anti-tumor effect and mechanism of ASPA and ASP are worthy to be studied since *H. diffusa* is widely used for treating tumors. Additionally, ASPA and ASP were useful markers for the quality control of *H. diffusa* and its herbal formula, and even the other plant materials containing these two compounds. In view of the results achieved in vitro, the in vivo anti-inflammatory effects and mechanisms of ASP and ASPA require further elucidation in a further study. Importantly, for the development of ASPA and ASP, research on the distribution in tissues, pharmacokinetic properties, and the safety after administration is necessary.

## 4. Materials and Methods

### 4.1. Chemicals, Reagents, and Cell Line

Asperulosidic acid (PubChem CID: 11968867, ASPA), asperuloside (PubChem CID: 84298, ASP), desacetyl asperulosidic acid (PubChem CID: 12315349, DAA), scandoside methyl ester (PubChem CID: 442433, SME), and *E*-6-*O*-*p*-coumaroyl scandoside methyl ester (PubChem CID: 44584784, CSME) were purchased from Shanghai Yuanye Biotechnology Co., Ltd. (Shanghai, China). Dulbecco’s Modified Eagle’s Medium (DMEM) and fetal bovine serum (FBS) were purchased from Gibco (Thermo Scientific, Waltham, MA, USA). The Cell Counting Kit-8 (CCK-8) was purchased from Dojindo (Kumamoto, Japan). LPS obtained from *Escherichia coli* O111:B4 was purchased from Sigma-Aldrich Co. (St. Louis, MO, USA). The antibodies against iNOS, COX-2, IκB-α, p38, and Erk1/2 were obtained from Proteintech Group, Inc. (Chicago, IL, USA). The antibodies against p-IκB-α, p-p38, p-Erk1/2, SAPK/JNK, and p-SAPK/JNK were purchased from Cell Signaling Technology, Inc. (Danvers, MA, USA). The ELISA kits for IL-6, IL-1β, and TNF-α were purchased from Neobioscience Technology Company (Shenzhen, China). The NO ELISA and bicinchoninic acid (BCA) assay kits were purchased from Beyotime Biotechnology (Shanghai, China). The PGE_2_ ELISA kit was from Enzo Life Sciences (New York, NY, USA). RAW 264.7 murine macrophages were purchased from the Cell Bank of the Chinese Academy of Science (Shanghai, China). All other reagents were of an analytical grade.

### 4.2. Cell Culture

RAW 264.7 cells were cultured in DMEM supplemented with 10% FBS, 100 units/mL penicillin, and 100 μg/mL streptomycin, and maintained in a carbon dioxide incubator (Thermo Fisher Scientific, Waltham, MA, USA) at 37 °C in 5% CO_2_ with a humidified atmosphere of 95% air. RAW 264.7 cells were induced with LPS (50 ng/mL) for 24 h after being incubated with iridoids for 1 h [16].

### 4.3. Cell Viability Assay

The cytotoxicity of the five iridoids on RAW 264.7 cells was measured using the CCK-8 assay. After being incubated for 24 h in 96-well plates at a density of 1 × 10^4^ cells/well, the cells were treated with serial concentrations of five iridoids (0, 50, 100, and 200 μg/mL) in triplicate for 1 h, and then induced with 50 ng/mL LPS for 24 h. Finally, 10 μL of CCK-8 was added to each plate and incubated for 1 h at 37 °C. The absorption wavelength was read at 450 nm using a Tecan microplate reader (Tecan Group Ltd., Männedorf, Switzerland). The cell viability was evaluated by comparing the absorbance values between the treatment groups and the control group, which was considered as 100%.

### 4.4. ELISA Assay of NO, PGE_2_, TNF-α, and IL-6

Briefly, after being incubated for 24 h at a density of 1 × 10^4^ cells/well in 96-well plates, RAW 264.7 cells were pretreated with five iridoids (0, 40, 80, and 160 μg/mL) in triplicate for 1 h. Then, the cells were induced with 50 ng/mL LPS for 24 h. Subsequently, the cell supernatant was collected and the concentration of NO, PGE_2_, TNF-α, and IL-6 was measured according to the ELISA manufacturer’s instructions using a Tecan microplate reader. The absorption wavelengths were set at 540, 450, 450, and 450 nm for NO, PGE_2_, TNF-α, and IL-6, respectively.

### 4.5. Real-Time PCR Assay

RAW 264.7 cells were incubated at 37 °C for 24 h in six-well plates at a density of 2 × 10^5^ cells/well. The cells were treated with ASP and ASPA at three concentrations (40, 80, and 160 μg/mL) for 1 h and induced with LPS (50 ng/mL) for 24 h. Total RNA was extracted from the RAW 264.7 cells using the RNAprep Pure Cell kit (Qiagen, Valencia, CA, USA) according to the manufacturer’s protocol. RNA (3 μL) concentration and purity was measured by the ratio of 260/280 nm absorbance and the remaining RNA solution was stored at −80 °C for reverse transcription. The total RNA was converted into the first-strand complementary DNA (cDNA) with a reverse transcription system as follows: 4 μL 5x prime Script RT Master MIX (Takara Bio INC., Kusatsu, Japan), 0.5 μg total RNA, and RNase-free water in a 20-μL reaction. The cDNA was used for real-time PCR (RT-PCR) and the reaction system contained 10 μL SYBR Premix EX Taq (2x) (Promega Corporation, Madison, USA), 1 μL forward primer (10 μM), 1 μL reverse primer (10 μM, iNOS, TNF-α, IL-6, and COX-2; Table 1), and 8 μL cDNA, which was amplified in an Applied Biosystems 7500 Fast Real-time PCR System version v2.3 (Thermo Fisher Scientific, Waltham, MA, USA) under the following reaction conditions: 50.0 °C for 3 min and 95.0 °C for 3 min, followed by 40 cycles of 95.0 °C for 10 s and 60.0 °C for 30 s. The threshold cycle (*C*_t_) was analyzed by the instrument’s software and fold changes in the mRNA expression were calculated according to the comparative *C*_t_ method (2^−ΔΔ*C*t^).

### 4.6. Western Blot Analysis

RAW 264.7 cells were incubated at 37 °C for 24 h in six-well plates at a density of 2 × 10^5^ cells/well. The cells were treated with ASP and ASPA at three concentrations (40, 80, and 160 μg/mL) for 1 h and induced with LPS (50 ng/mL) for 24 h. Subsequently, 100 μL cell lysis buffer (10 mM Tris-HCl, 0.15 M NaCl, 5 mM ethylenediaminetetraacetic acid (EDTA), 1% Triton×100, 5 mM dithiothreitol (DTT), and 0.1 mM phenylmethanesulfonyl fluoride (PMSF)) was added and incubated for 30 min at 4 °C and then centrifuged at 12,000 rpm for 10 min to collect the supernatant for protein analysis using the BCA assay kit after adding 5× loading buffer for Western blotting. All protein samples were loaded onto 12% sodium dodecyl sulfate-polyacrylamide gel electrophoresis (SDS-PAGE) and transferred onto a polyvinylidene fluoride (PVDF) membrane. The membrane was blocked for 2 h with 10% non-fat milk at room temperature and then incubated overnight with the primary antibodies, including those against iNOS (1:1000), COX-2 (1:1000), IκB-α (1:1000), p-IκB-α (1:1000), p38 (1:1000), p-p38 (1:1000), Erk1/2 (1:1000), p-Erk1/2 (1:1000), SAPK/JNK (1:1000), p-SAPK/JNK (1:1000), and β-Actin (1:1000), at 4 °C. The membranes were washed three times and incubated with the secondary antibody (1:10,000) at room temperature for 1 h. Finally, the blots were measured by an ECL chemiluminescence method and a Western blotting detection System (FluorChem R, ProteinSimple, San Jose, CA, USA).

### 4.7. Statistical Analyses

All experiments were performed in triplicate. Data were analyzed using IBM SPSS Statistics 20.0 (IBM SPSS Statistics, Chicago, IL, USA) and are presented as the mean ± standard deviation (SD). One-way ANOVA followed by Tukey’s multiple comparison test was used to assess the statistical differences among groups. *p* < 0.05 was considered significant.

## 5. Conclusions

The present study compared the anti-inflammatory effects of the five iridoids found in *H. diffusa* and shows that ASP and ASPA have an anti-inflammatory effect. ASP and ASPA could significantly decrease the levels of PGE_2_, NO, TNF-α, and IL-6 in LPS-induced RAW 264.7 cells. The possible mechanism involved the down-regulation of the expressions of inflammatory mediators and pro-inflammatory cytokines via the inhibition of NF-κB and MAPK signaling pathways. Taken together, ASP and ASPA may be potent bioactive iridoids to treat inflammatory diseases.

## Figures and Tables

**Table 1:** The primers used for RT-PCR analysis.

Genes	Sense Primer Sequence 5′–3′	Antisense Primer Sequence 5′–3′
*TNF-α*	GCGACGTGGAACTGGCAGAA	CAGTAGACAGAAGAGCGTGGTG
*IL-6*	GTTGCCTTCTTGGGACTGAT	CATTTCCACGATTTCCCAGA
*iNOS*	TGGAGCGAGTTGTGGATTGT	CTCTGCCTATCCGTCTCGTC
*COX-2*	ACCTGGTGAACTACGACTGC	TGGTCGGTTTGATGTTACTG
*β-actin*	TGCTGTCCCTGTATGCCTCTG	GCTGTAGCCACGCTCGGTCA
